# Supplementary figures and images for: Tracking a serial killer: Integrating phylogenetic relationships, epidemiology, and geography for two invasive meningococcal disease outbreaks
Source: PLoS One. 2018 Nov 28;13(11):e0202615. doi: 10.1371/journal.pone.0202615 (PMC6261407; doi:10.1371/journal.pone.0202615)

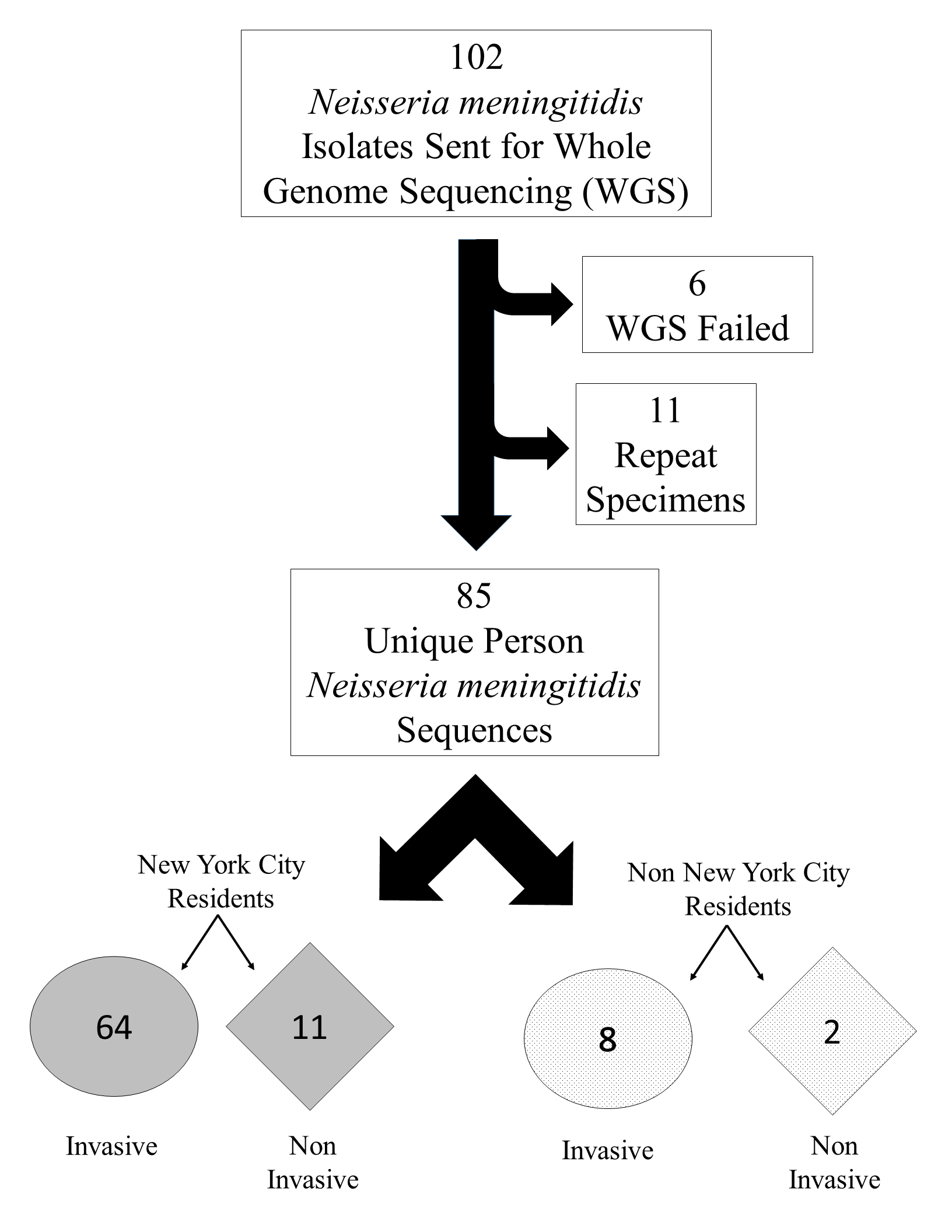

Supplement: S1 Fig — (TIF) [file pone.0202615.s001.tif]
